# CD8+/FOXP3+ ratio and PD-L1 expression associated with survival in pT3N0M0 stage esophageal squamous cell cancer

**DOI:** 10.18632/oncotarget.12213

**Published:** 2016-09-23

**Authors:** Yingming Zhu, Minghuan Li, Dianbin Mu, Li Kong, Jianbo Zhang, Fen Zhao, Zhenxiang Li, Xuemei Liu, Cong Bo, Jinming Yu

**Affiliations:** ^1^ Department of Radiation Oncology, Shandong Cancer Hospital and Institute, Shandong University, Jinan, China; ^2^ Department of Pathology, Shandong Cancer Hospital and Institute, Shandong University Jinan, China

**Keywords:** esophageal squamous cell cancer, CD8, forkhead box protein 3, programmed death receptor ligand-1, pT3N0M0

## Abstract

Data describing relationships between the tumor immune microenvironment and patient outcome are limited for esophageal squamous cell cancer (ESCC). The present study investigated the prognostic values of programmed death-ligand 1 (PD-L1) expression and CD8+ or forkhead box protein 3+ (FOXP3+) tumor-infiltrating lymphocytes (TILs) in 133 pathological T3N0M0 stage ESCC patients who underwent radical resection without neoadjuvant or adjuvant therapy. CD8+ and FOXP3+ TIL densities as well as PD-L1 levels in tumor cells and lymphocytes, were assessed through immunohistochemical staining. Patient survival was not associated with CD8+ or FOXP3+ TILs alone, but PD-L1 expression and the CD8+/FOXP3+ ratio were independent predictors of both disease-free and overall survival. PD-L1 expression correlated with age (*p* = 0.029), tumor length (*p* < 0.001), tumor differentiation status (*p* = 0.002) and reduced intratumoral CD8+ TIL density (*p* < 0.001). Our results suggest pT3N0M0 ESCC clinical outcomes correlate with CD8+ and FOXP3+ TIL densities and PD-L1 levels. Moreover, an intrinsic mechanism for induction of PD-L1 overexpression may be occurring during early tumor oncogenesis. This information may be useful for stratifying patients and guide the application of checkpoint blockade therapy in ESCC.

## INTRODUCTION

Esophageal squamous cell carcinoma (ESCC) remains the predominant type of esophageal cancer (EC) in the “EC Belt”, which extends from China via central Asia to northern Iran [1]. Despite significant advances in screening, diagnosis and treatment modalities, ESCC patient prognosis remains poor. Clinical outcome for patients with middle range ESCC (generally stage IB, II, and IIIA) can vary significantly, with some achieving long-term survival, while others die following disease recurrence [2, 3]. Novel ESCC-specific molecular targets are therefore needed to improve prognosis prediction and therapeutic interventions.

Patients with pathological T3N0M0 stage (pT3N0M0) disease represent a considerable fraction of middle-stage patients. Although they have the same TNM stage, they are divided into stages IB, IIA, and IIB based on tumor location and histologic grade in the 7th edition of the AJCC manual [4]. However, the classic AJCC/UICC-TNM system only describes tumor burden on a macro scale, and does not provide detailed information on the microenvironment in which ESCC proliferates. Considerable research has focused on the host immune response against cancer and illuminated the prognostic importance of tumor-infiltrating lymphocytes (TILs) in many tumor types [5]. However, discussions of TILs with respect to ESCC have been limited and somewhat inconsistent. While one study observed improved prognosis in patients with abundant CD8+ TILs, another found no association between patient survival and CD8+ TILs in residual tumor or scar tissues [6, 7]. Inconsistent results concerning regulatory T cells (Tregs), defined in this study as forkhead box protein 3 (FOXP3)+ T cells (FOXP3+ TILs), have also been reported in ESCC [6, 8]. Still, multiple studies associated TILs with clinicopathological factors such as survival, response to neoadjuvant therapy and even tumor stage in ESCC [6, 7, 9]. Along with TIL densities, TIL-tumor cell interactions have been increasingly emphasized.

As an immune checkpoint, the programmed death-1 (PD-1)/programmed death-ligand 1 (PD-L1) axis facilitates T-cell exhaustion and enables tumors to avoid immunosurveillance [10]. PD-L1-PD-1 pathway blockade improves overall survival (OS) in non-small-cell lung cancer (NSCLC), urothelial carcinoma, melanoma, and renal cell carcinoma patients [11–14]. Thus far, no convincing data has been presented regarding the efficacy of anti-immune checkpoint agents in ESCC. Taube *et al*. proposed stratifying tumors according to PD-L1 status, and TIL presence or absence in the microenvironment might predict patient response to anti-PD-1 therapy [15]. However, interactions between PD-L1 and different TIL subpopulations in ESCC, as well as associated clinical outcomes, are under-studied. In the present study, we investigated CD8+ or FOXP3+ TIL (Treg) densities and PD-L1 expression in a cohort of 133 resected pT3N0M0 ESCC patients without neoadjuvant and adjuvant therapy. We speculate on potential mechanisms for PD-L1 overexpression and assess the impacts of CD8+ and/or FOXP3+ TIL densities and PD-L1 levels on patient survival.

## RESULTS

### Patient characteristics

133 patients with pathological T3N0M0 stage ESCC who underwent radical Ivor-Lewis esophagetomies were included in this study (Table 1). 75 patients (56.4%) were male and 58 (43.6%) were female, with a median age of 59 years. 110 patients (82.7%) had recurrence and 95 (71.43%) died during the follow-up period. The estimated 1-, 3- and 5-year DFS and OS rates were 87%, 47% and 26%, and 96%, 61% and 38%, respectively. Median DFS and OS were 33.7 and 42.6 mo, respectively.

**Table 1:** Correlation of PD-L1 expression with clinicopathological parameters

	No. patients	%	Intratumoral PD-L1 expression		
			Negative	Positive	*P* value
Age (years)					**0.029**
≤ 59	67	50.4%	45	22	
> 59	66	49.6%	32	34	
Gender					0.361
Male	75	56.4%	46	29	
Female	58	43.6%	31	27	
Pre-op KPS					0.188
KPS ≤ 80	89	66.9%	48	41	
KPS > 80	44	33.1%	29	15	
Tumor location					0.801
Upper	23	17.3%	12	11	
Middle	57	42.9%	33	24	
Lower	53	39.8%	32	21	
Tumor length					**< 0.001**
≤ 4 cm	70	52.6%	51	19	
> 4 cm	63	47.4%	26	37	
Differential Grade					**0.002**
Well	46	34.6%	32	14	
Moderate	53	39.8%	34	19	
Poor	34	25.6%	11	23	
Histological Type					0.372
Ulcerative type	68	51.1%	36	32	
Medullary type	47	35.3%	31	16	
Fungating type	18	13.5%	10	8	
Recurrence					**0.037**
Yes	110	82.7%	59	51	
No	23	17.3%	18	5	

KPS, Karnofsky performance score; PD-L1, programmed death-ligand 1.

### Analysis of immunohistochemical parameters

Stromal infiltration was more frequently observed than diffuse infiltration, which leads to more abundant cells in the stroma than the cancer nest (Figure S1). Immune cell infiltration was evaluated, excluding hemorrhagic, necrotic and fibrotic areas. The CD8+/FOXP3+ TILs ratio varied substantially among samples. The median intratumoral CD8+ or FOXP3+ TIL densities, and CD8+/FOXP3+ TILs ratio were 61.44 cells/HPF, 23.66 cells/HPF, and 2.64, respectively (Table S1). CD8+ TILs and FOXP3+ TILs were moderately correlated (*r*, 0.534; *p* < 0.001; Figure 2A).

**Figure 1:**
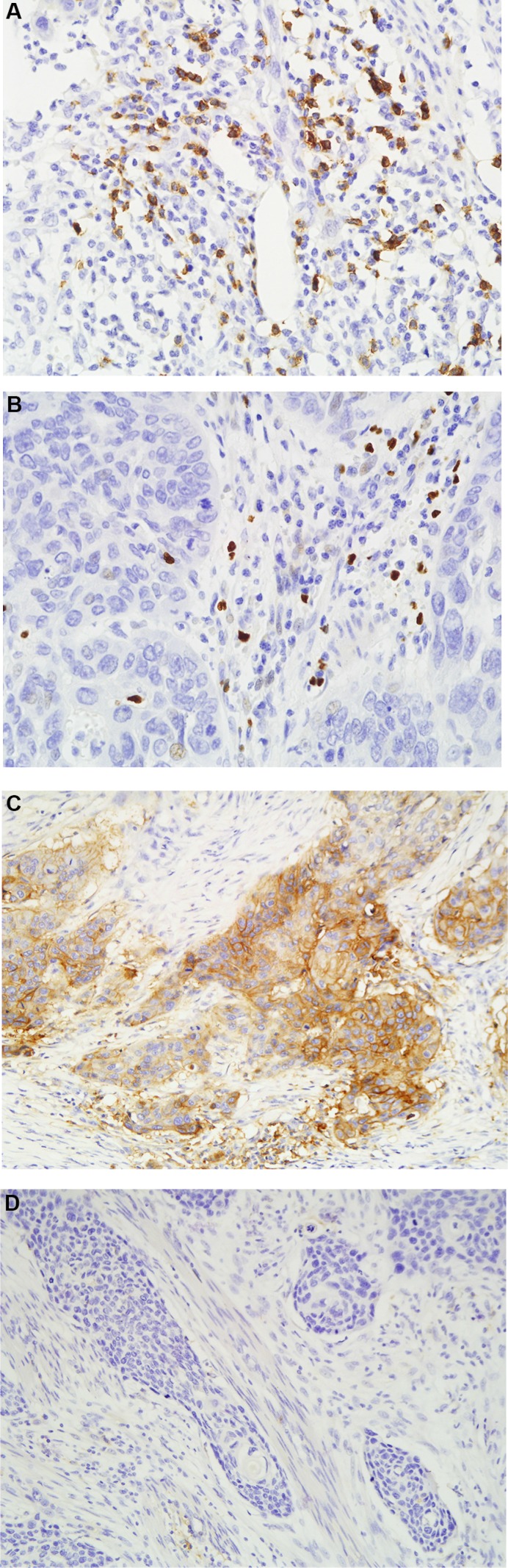
Representative positive CD8 (**A**) and FOXP3 (**B**) staining (×400 magnification), and positive (**C**) and negative (**D**) PD-L1 expression (×200 magnification) in ESCC samples.

**Figure 2:**
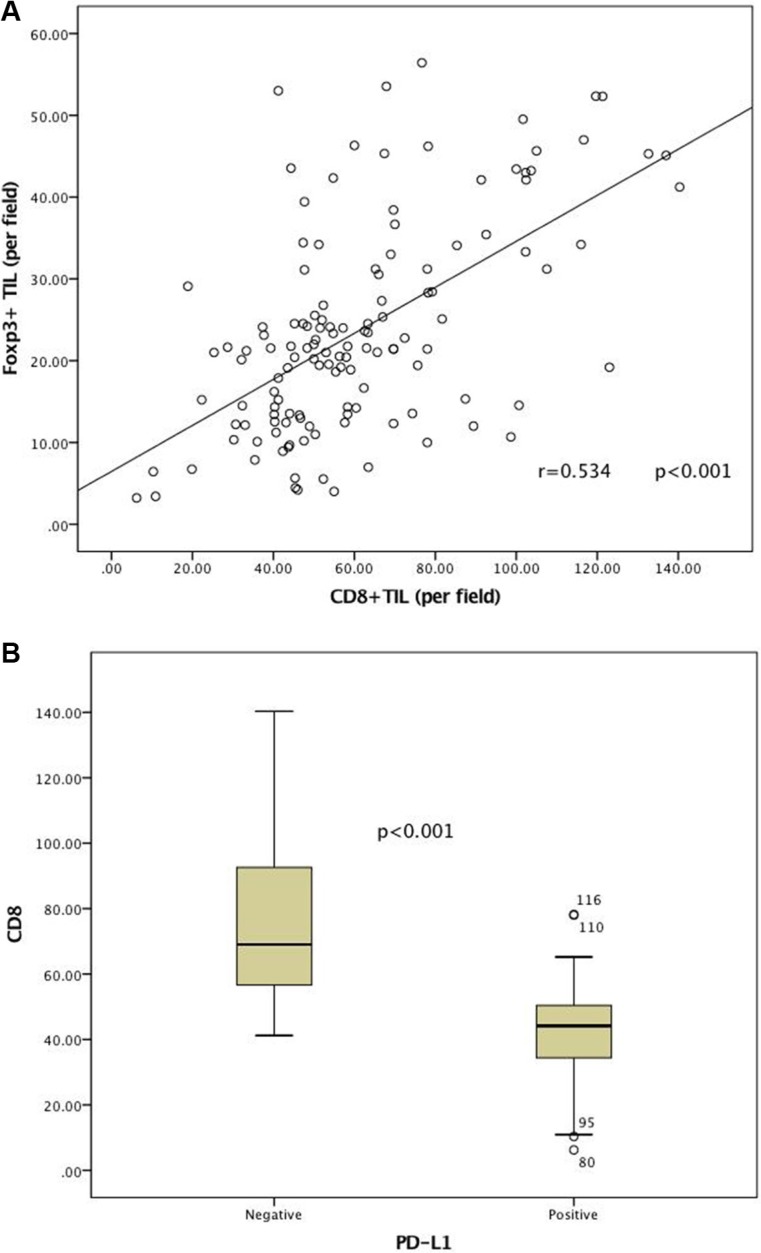
Correlation between immune factors Strong positive correlations were observed between CD8+ or FOXP3+ TILs infiltration (**A**) CD8+ TILs densities with respect to PD-L1 expression (**B**).

PD-L1 showed a predominantly membranous or cytoplasmic (or both) focal or scattered staining pattern (51.12% of all specimens, Figure 1C) and was detected in tumor cells and in some infiltrating lymphocytes (staining in either cell type was considered positive). A strong positive correlation and concordance was observed in PD-L1 expression (positive and negative expression) detected using immunohistochemistry (IHC) (*r* = 0.853, *p* = 0.002; Table S2). PD-L1 expression and CD8+ TIL densities (*r* = −0.67, *p* < 0.001) were negatively correlated (Table S2; Figure 2B). Patients with positive PD-L1 expression exhibited reduced FOXP3+ TIL infiltration and lower CD8+/FOXP3+ TILs ratios; however, these associations were not statistically significant (Table S3).

### Immunohistochemical and clinicopathological parameters correlations

Positive tumor-related PD-L1 expression was correlated with older age (> 59, *p* = 0.029), longer tumor length (> 4 cm, *p* < 0.001), poorer histological differentiation (*p* = 0.002) and tumor recurrence (*p* = 0.037) (Table 1; Figure S2). There was no relationship found between CD8+ or FOXP3+ TIL densities, or CD8+/FOXP3+ TILs ratio and clinicopathological variables (Table S4).

### Prognostic factors

**Figure 3:**
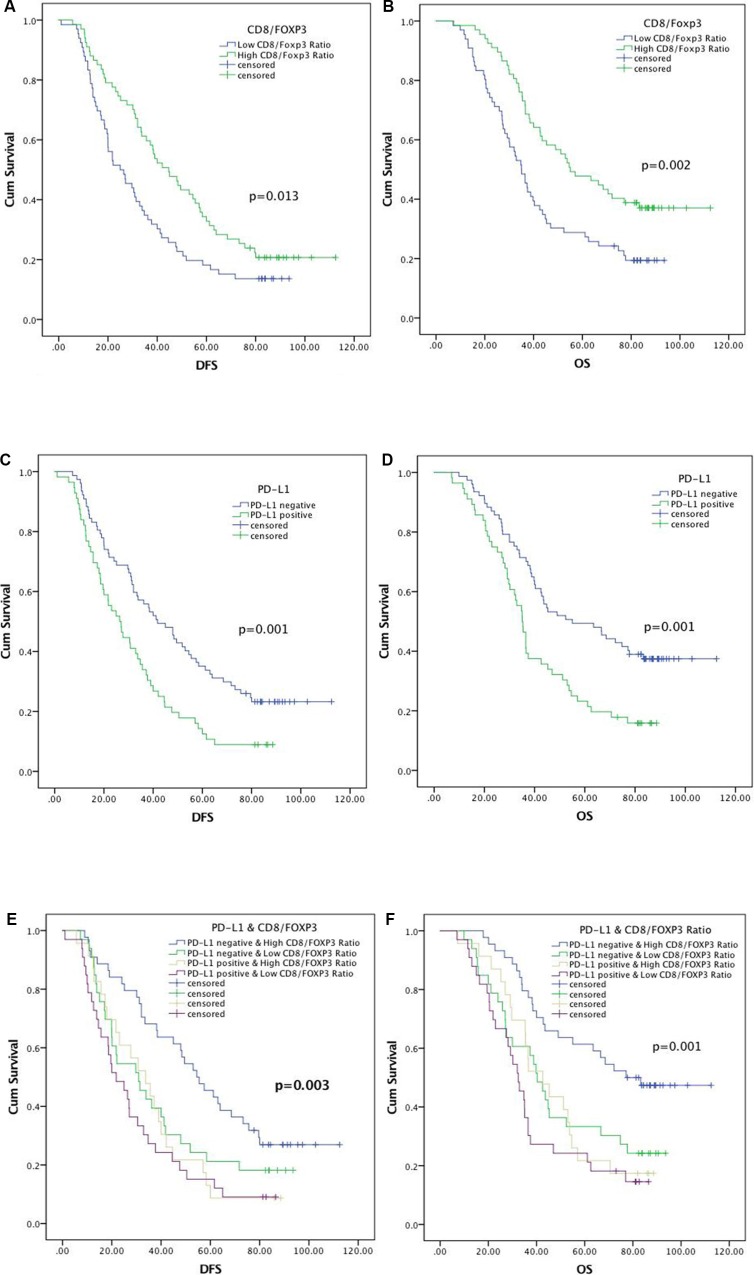
Kaplan–Meier analysis of DFS and OS for CD8+/FOXP3+ TILs ratio (**A**) and (**B**), PD-L1 expression (**C**) and (**D**), and relationship between CD8+/FOXP3+ TILs ratio and PD-L1 expression (**E**) and (**F**).

Neither FOXP3+ nor CD8+ TILs alone was related to patient prognosis in the Kaplan-Meier analysis (Table 2). In contrast, CD8+/FOXP3+ TILs ratio and PD-L1 expression were correlated with both DFS (*p* = 0.013, 0.001 respectively; Figure 3A and 3C; Table 2) and OS (*p* = 0.002, 0.001 respectively; Figure 3B and 3D; Table 2) in univariate analyses. Patients with high CD8+/FOXP3+ TILs ratios and negative PD-L1 expression experienced better DFS and OS compared to the other three groups (Figure 3E–3F). Because PD-L1 expression was associated with tumor differentiation and length, we divided patients into well and moderately/poorly differentiated subgroups and adjusted for tumor length (≥ 4 cm or < 4 cm). In both well and moderately/poorly differentiated subgroups with similar tumor lengths, patients with positive PD-L1 expression had poorer DFS (*p* = 0.043 and 0.022, respectively) and OS (*p* = 0.009 and 0.032, respectively) compared to patients with negative PD-L1 expression (Figure S3A–S3D and S4A–S4D). Moreover, cox regression analyses demonstrated that both PD-L1 expression and CD8+/FOXP3+ TILs ratio were independent predictors of DFS (HR 1.596, 95% CI 1.086-2.343, *p* = 0.017; HR 0.843, 95% CI 0.748-0.951, *p* = 0.005; respectively) and OS (HR 1.730, 95% CI 1.147–2.609, *p* = 0.009; HR 0.796, 95% CI 0.688–0.920, *p* = 0.002; respectively) (Table 3). Preoperative Karnofsky performance score (KPS) was also an independent prognostic factor (*p* = 0.049 for OS, *p* = 0.038 for DFS, Table 3). Tumor differentiation was associated with DFS in univariate analysis (*p* = 0.046) but was not an independent prognostic factor in multivariate analyses (*p* = 0.108; Tables 2 and 3). Age, gender, tumor location, tumor length and histological type were not associated with ESCC patient prognosis in this study (Table 2).

**Table 2:** Univariate analysis of clinicopathological and IHC parameters associated with DFS and OS

Variable	DFS		OS	
	HR (95% CI)	*P* value	HR (95% CI)	*P* value
Age (years) (< 59.5 vs. ≥ 59.5)	1.046 (0.719–1.521)	0.816	1.033 (0.691–1.545)	0.874
Gender (male vs. female)	1.203 (0.824–1.755)	0.338	1.241 (0.828–1.860)	0.295
Pre-op KPS (≤ 80 vs. > 80)	0.622 (0.413–0.936)	**0.023**	0.607 (0.388–0.949)	**0.029**
Tumor location				
Overall		0.502		0.136
Upper vs. Middle	0.827 (0.489–1.399)	0.479	0.956 (0.546–1.673)	0.875
Upper vs. Lower	0.730 (0.430–1.239)	0.243	0.632 (0.353–1.131)	0.122
Tumor length (≤ 4 cm vs. > 4 cm)	1.099 (0.755–1.598)	0.623	1.143 (0.764–1.711)	0.515
Differential Grade				
Overall		**0.046**		0.095
Well vs. Moderate	1.394 (0.890–2.183)	0.147	1.396 (0.859–2.270)	0.179
Well vs. Poor	1.847 (1.136–3.005)	**0.013**	1.785 (1.055–3.020)	**0.031**
Well vs. Moderate + Poor	1.552 (1.034–2.330)	**0.034**	1.535 (0.986–2.388)	0.057
Histological Type				
Overall		0.739		0.872
Ulcerative type vs. Medullary type	0.851 (0.563–1.287)	0.445	0.908 (0.582–1.415)	0.668
Ulcerative type vs. Fungating type	0.979 (0.555–1.728)	0.943	1.504 (0.572–1.940)	0.866
IHC markers				
CD8+ TILs (low vs. high)	0.934 (0.642–1.358)	0.720	0.868 (0.580–1.300)	0.493
FOXP3+ TILs (low vs. high)	1.319 (0.907–1.918)	0.147	1.317 (0.880–1.972)	0.180
CD8+/FOXP3+ TILs Ratio (low vs. high)	0.622 (0.427–0.907)	**0.014**	0.534 (0.356–0.802)	**0.003**
Intratumoral PD-L1 expression (negative vs. positive)	1.845 (1.262–2.698)	**0.002**	1.957 (1.303–2.939)	**0.001**

DFS, disease-free survival; OS, overall survival; KPS, Karnofsky performance score; TILs, tumor-infiltrating lymphocytes; PD-L1, programmed death-ligand 1.

**Table 3:** Multivariate analysis of clinicopathological and immunohistochemical parameters associated with DFS and OS

Factor	Median Survival		Hazard Ratio (95% CI)	*P* value
OS				
Pre-op KPS			0.975 (0.951–1.000)	0.054
CD8+/FOXP3+ TILs Ratio			0.796 (0.688–0.920)	**0.002**
Intratumoral PD-L1 expression (negative vs. positive)	55.0	35.1	1.730 (1.147–2.609)	**0.009**
DFS				
Pre-op KPS			0.977 (0.954–1.001)	0.057
Differential Grade (Well vs. Moderate + Poor)	37.2	32.0	1.404 (0.928–2.123)	0.108
CD8+/FOXP3+ TILs Ratio			0.843 (0.748–0.951)	**0.005**
Intratumoral PD-L1 expression (negative vs. positive)	41.8	26.4	1.596 (1.086–2.343)	**0.017**

DFS, disease-free survival; OS, overall survival; KPS, Karnofsky performance score; TILs, tumor-infiltrating lymphocytes; PD-L1, programmed death ligand 1.

## DISCUSSION

Our study assessed the ESCC tumor microenvironment with respect to tumor cells and TILs in 133 pT3N0M0 patients who had received radical surgery. We demonstrated a strong negative correlation between PD-L1 expression and CD8+ TILs, and correlated PD-L1 expression and CD8+/FOXP3+ TILs ratio with DFS and OS. A patient subgroup with both negative PD-L1 expression and a high CD8+/FOXP3+ TILs ratio tended to show improved survival and reduced recurrence. Previous studies of the ESCC tumor microenvironment have tended to evaluate TILs and tumor PD-L1 expression separately, without regard to combined prognostic values.

A higher CD8+/FOXP3+ TILs ratio has been related to favorable prognosis in aggressive breast cancer, ovarian cancer and osteosarcoma [16–18]. Similarly, we observed that the prognostic value of this ratio is better than that of FOXP3+ or CD8+ TILs alone, which partly reflects reciprocal interactions between promotive CD8+ TILs and repressive FOXP3+ TILs (Tregs) in tumors [19, 20]. Thus, assessment of both FOXP3+ and CD8+ TIL densities may provide more detailed information about patient immune state.

The use of different antibody clones for IHC may explain recent contradictory reports associating PD-L1 expression with solid tumor patient prognoses. In our study, we used one of four antibody clones approved by the US Food and Drug Administration (FDA) for PD-L1 IHC detection (SP142) and validated our results via qRT-PCR in 10 tissue samples [21]. We assessed PD-L1 expression in both tumor cells and stromal lymphocytes and found that PD-L1 was correlated with postoperative recurrence and poor patient survival. This is in agreement with previous studies that only evaluated PD-L1 expression in tumor cells [22–24]. Therefore, these clinical data provide rationale for inhibiting PD-L1 in ESCC patients. In our study, poorly differentiated tumors longer than 4 cm displayed stronger PD-L1 expression than well and moderately differentiated tumors of shorter length. Consistent with our results, other studies have reported a correlation between PD-L1 expression and higher tumor grade in various cancers, although the functions of PD-L1 in tumor differentiation and growth were not addressed [25–29]. It might be related to the slightly worse hypoxia conditions in longer tumors, as PD-L1 is reportedly induced under hypoxia conditions [30, 31]. Additionally, upstream molecules involved in PD-L1 expression may also promote cellular proliferation [27, 32]. Another possible explanation is that activation of the PD-1/PD-L1 pathway may promote tumor immune escape, allowing tumor cells to proliferate and spread more rapidly [28].

Molecular mechanisms underlying immune avoidance appear to be distinct among tumors. To date, two phenotypes have been identified: T cell-inflamed (also known as adaptive immune resistance) and non-T cell-inflamed (also known as innate immune resistance) [33]. The two phenotypes are differentiated by whether PD-L1 upregulation is driven by constitutive oncogenic signaling pathways or induced in reaction to inflammatory signals that are produced when antitumor immune responses are activated [33]. Our study observed a moderate negative association between PD-L1 and CD8+ TILs, which suggested that intrinsic pathways might prevail over inflammatory signals, drive PD-L1 overexpression and block tumor immunity in early ESCC. Evidence suggests that patient response to single-agent PD-1 blockade would be less effective in the setting of innate immune resistance as compared to adaptive immune resistance [34, 35]. Further studies are required to elucidate PD-L1 overexpression mechanism(s) within the tumor microenvironment and the relevance of such mechanisms to ESCC patient outcome following PD-1 blockade.

Our study had several limitations. First, while pT3N0M0 stage ESCC patient optimal management strategies are still controversial, surgery alone is not standard treatment for such patients. We analyzed tumor original immune states, which are not affected by chemotherapy and radiotherapy. However, preoperative chemoradiotherapy may alter the tumor immune microenvironment, inducing T cell influx and PD-L1 upregulation, and this requires further study [23, 36, 37]. Second, we did not analyze TILs and PD-L1 expression in different subareas of the tumor microenvironment, such as invasive margin, cancer cell nests or peritumoral sites. Third, while we examined five tumor cores per patient from tissue regions that were considered representative, we acknowledge that tissue microarrays have limitations for analysis of tumor heterogeneity. Further studies are warranted to harmonize and standardize testing for CD8, FOXP3 and PD-L1 expression by IHC in larger samples.

Within the context of these limitations, our study identified an inverse relationship between CD8+ TILs and PD-L1 expression, suggesting that an intrinsic induction-type mechanism is active in ESCC. Our findings also suggest that differences in TIL density and PD-L1 expression may partly explain the diversity of clinical outcomes observed in patients with similar ESCC stages. These findings may be suggestive of the optimal treatment choice and checkpoint blockade therapy in patients with pT3N0M0 ESCC.

## MATERIALS AND METHODS

### Patients and specimens

The study cohort included 133 patients with pathological T3N0M0 stage thoracic ESCC who had radical esophagectomies in Shandong Cancer Hospital and Institute since June 2005. No patient exhibited evidence of distant metastases in preoperative examination, none had received any prior anticancer treatments or postoperative adjuvant therapy, and all underwent complete macroscopic tumor removal. All tumor tissues were confirmed as ESCC by hematoxylin and eosin (H&E) staining after surgical resection. Only patients who survived more than three mo post-surgery were included in the study. Tumor stage was determined according to the American Joint Committee on Cancer (AJCC, 2009) TNM (tumor, node, metastases) staging system [4]. Each tumor was catergorized histologically according to WHO classification criteria. Follow-ups were completed December 2015. The median follow-up time was 42.6 mo. Overall survival (OS) was defined as the time between surgery and death or last known follow-up. The study design was approved by the Ethics Committee of Shandong Cancer Hospital, and all patients signed informed consent agreements.

### Immunohistochemistry

IHC analyses were performed using the streptavidin-biotin-peroxidase method with postoperative specimens from all patients. 4-μm thin sections were deparaffinized with xylene, rehydrated with graded alcohol solutions, and then exposed to the antigen retrieval system under high pressure for two min. Sections were stained with anti-CD8 (clone SP16, Beijing Zhongshan Golden Bridge Biotechnology Company, Beijing, China), anti-FOXP3 (clone 236A/E7, ab20034, Abcam, USA) and anti-PD-L1 (clone SP142, Beijing Zhongshan Golden Bridge Biotechnology Company) in a humidified chamber at 37°C for 60 min. Slides were incubated with a secondary anti-rabbit and anti-mouse antibodies (Beijing Zhongshan Golden Bridge Biotechnology Company) at 37°C for 15 min. Diamino-benzidine (DAB) was then added to visualize CD8, FOXP3 and PD-L1 staining and slides were counterstained with hematoxylin.

### CD8, FOXP3 and PD-L1 evaluation and quantification

Quantitative evaluations of CD8+ or FOXP3+ TILs were performed by examining at least five non-overlapping high-power fields (40× objective and 10× eyepiece) with the most abundant TILs in each stained section. PD-L1 expression was considered positive if distinct membranous or cytoplasmic staining was observed in tumor or stromal cells. PD-L1 staining intensity was recorded as follows: negative for no staining, positive for membranous or cytoplasmic staining in tumor cells or lymphocytes. Sections were evaluated independently by two experienced pathologists blinded to clinicopathologic information. Variations of > 5% were reassessed and a consensus was reached. As other cell populations can stain with CD8 and FOXP3 antibodies, CD8+ or FOXP3+ cells with apparent different morphological appearances were excluded from cell counts. FOXP3+ and CD8+ TILs were counted both in the cancer cell nest and tumor stroma. The CD8+/FOXP3+ TILs ratio was defined as the number of CD8+ TILs divided by the number of FOXP3+ TILs (Tregs). The mean numbers of CD8+ TILs and FOXP3+ TILs per field and the ratio of CD8+/FOXP3+ TILs were calculated. ESCCs were classified into high and low groups for CD8+ TILs, FOXP3+ TILs and the CD8+/FOXP3+ TILs ratio using a cut-off value defined as the median number of infiltrating cells per field, as described previously (Figure 1) [7, 8]. The results are shown as the mean ± SE number of cells in one field (Table S1).

### RNA preparation, reverse transcription and quantitative RT-PCR

To validate the PD-L1 IHC detection method, PD-L1 mRNA was quantified via qRT-PCR in 10 tissue samples. Total RNA was extracted using the RNA extraction kit spin column method (Qiagen, Dusseldorf, Germany) following the manufacturer's introductions. Total RNA (5 ng) was reverse transcribed with cDNA Reverse Transcription kits (Toyobo Co., LTD, Osaka, Japan) according to the manufacturer's instructions. qPCR was conducted in a SYBR Green PCR Master Mix (ROX). Relative gene expression was calculated using the comparative cycle threshold (CT) (2^−ΔΔCT^) method, and normalized to with the endogenous control, GADPH. The following primers were used in this study: HIF-1α, 5′-GAACGTCGAAAAGAAAAGTCTCG-3′(forward) and 5′ -CCTTATCAAGATGCGAACTCACA-3′ (reverse); PD-L1, 5′ - TTGCTGAACGCCCCATACAA-3′ (forward) and 5′ - TCCAGATGACTTCGGCCTTG-3′ (reverse); and GADPH, 5′ - GCACCGTCAAGGCTGAGAAC-3′ (forward) and 5′ - TGGTGAAGACGCCAGTGGA-3′ (reverse).

### Statistical analyses

Statistical analyses were performed with SPSS version 19.0 (IBM SPSS, Chicago, Illinois, USA). Cumulative survival time was calculated using the Kaplan-Meier method and compared using the log-rank test. Univariate and multivariate analyses were performed according to the Cox proportional hazards model. Associations among variables were evaluated using Fisher's exact test or the χ^2^ tests, and Spearman's correlation tests were used to analyze possible associations. All statistical tests were two sided and results were considered significant when *p* ≤ 0.05.

## SUPPLEMENTARY MATERIALS FIGURES AND TABLES


