# Supplementary material for: CD8+/FOXP3+ ratio and PD-L1 expression associated with survival in pT3N0M0 stage esophageal squamous cell cancer
Source: Oncotarget. 2016 Sep 23;7(44):71455–65. doi: 10.18632/oncotarget.12213 (PMC5342092; doi:10.18632/oncotarget.12213)
Supplement: Supplementary file 1 [file oncotarget-07-71455-s001.pdf]

CD8+/FOXP3+ ratio and PD-L1 expression associated with survival in pT3N0M0 stage esophageal squamous cell cancer

Supplementary Materials

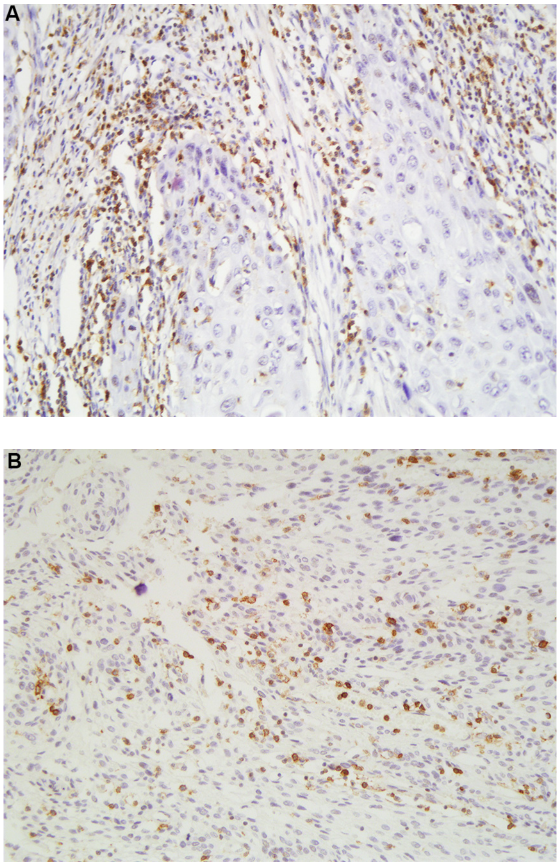

Supplementary Figure S1: Representative tumor tissues depicting CD8+ or FOXP3+ TIL localization patterns (×200 magnification). Two distinct T-lymphocyte infiltration patterns are predominantly found in ESCC: stromal (**A**) and diffuse (**B**) infiltration

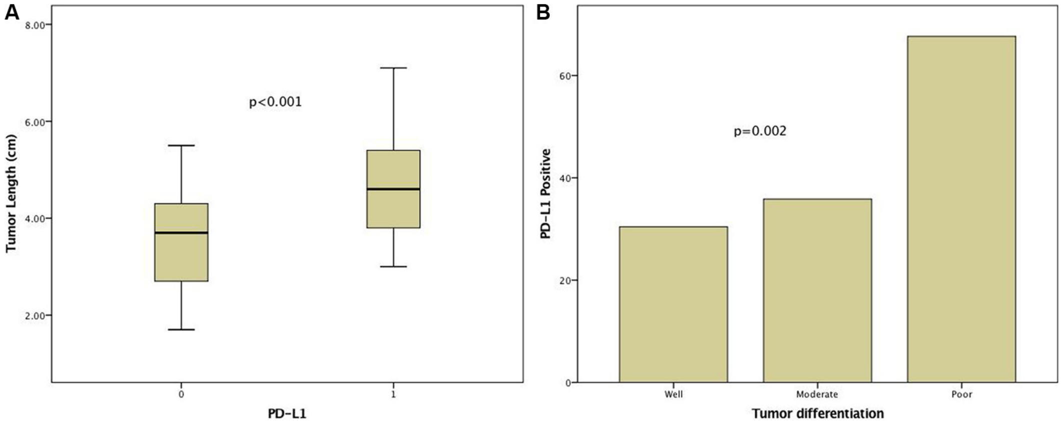

Supplementary Figure S2: Correlation between PD-L1 and clinicopathological parameters. Tumor lengths with respect to PD-L1 expression (**A**) Distribution of PD-L1 positive cases with respect to tumor differentiation (**B**)

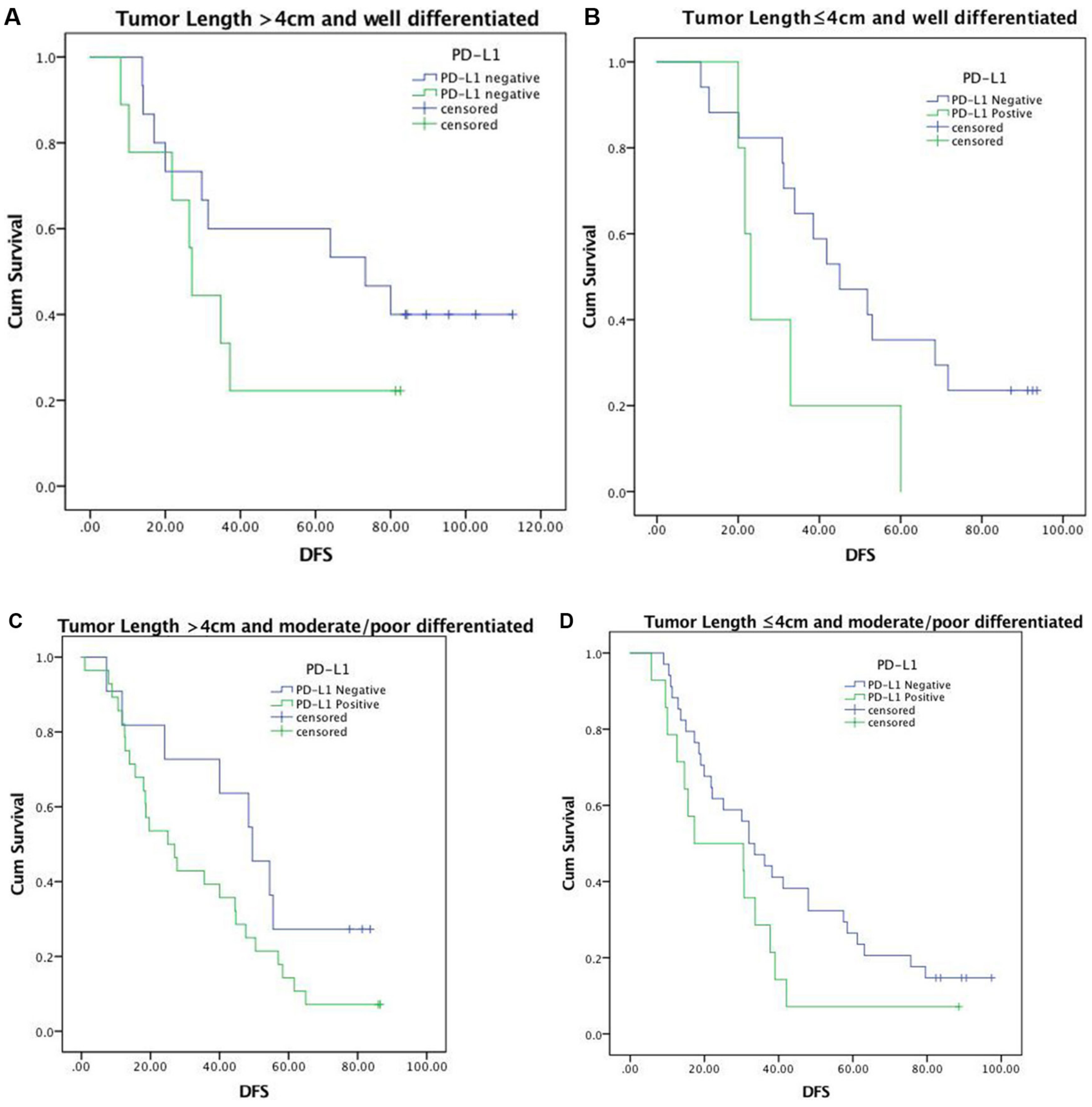

Supplementary Figure S3:Kaplan–Meier analysis of DFS in well. (**A**) and (**B**) and moderately/poorly differentiated ESCC (**C**) and (**D**), adjusted for tumor length.

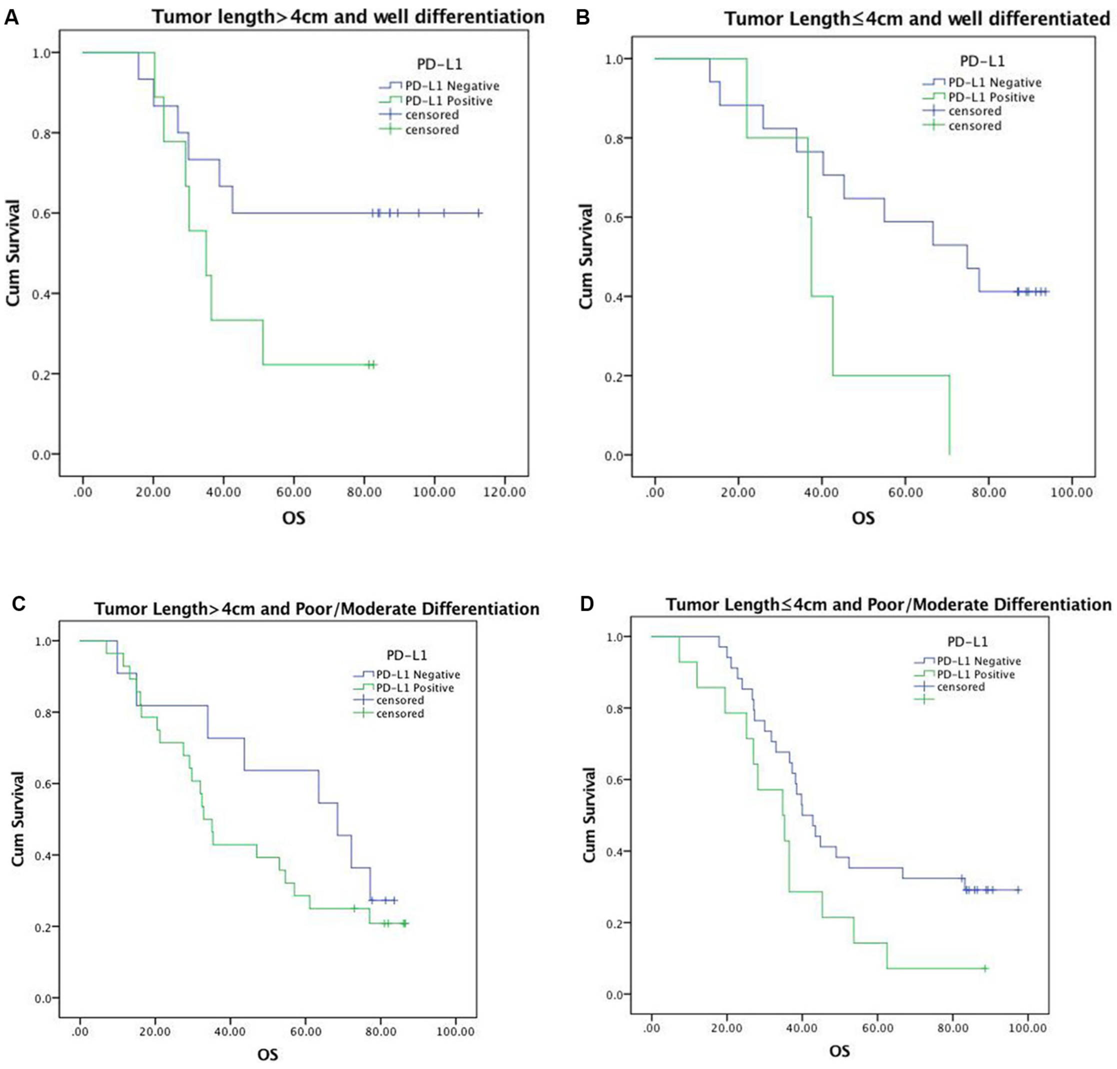

Supplementary Figure S4: Kaplan–Meier analysis of OS in well. (**A**) and (**B**) and moderately/poorly differentiated ESCC (**C**) and (**D**), adjusted for tumor length.

Supplementary Table S1: TILs in patients undergoing esophagectomy

Variables	Mean	Median	SE	Skewness	Interquartile Range
CD8+ TILs	61.44	55.00	26.74	0.83	44.17–74.99
FOXP3+ TILs	23.66	21.48	12.83	0.67	13.47–31.21
CD8+/FOXP3+ Ratio	3.19	2.64	2.09	2.46	2.07–3.46

CD8+ or FOXP3+ TILs are represented as counts/high-power field (×400 magnification).
ESCC, esophageal squamous cell cancer; TILs, tumor-infiltrating lymphocytes.

Supplementary Table S2: PD-L1 expression as measured by qRT-PCR and PD-L1 IHC score in 10 representative patients

Patient No.	PD-L1 mRNA expression relative to GADPH ($2^{-\Delta\Delta CT}$ method)	IHC signal
1	1.0119	+
2	1.1464	+
3	1.7150	+
4	0.8407	+
5	0.9379	+
6	0.8166	+
7	0.0011	−
8	0.0039	−
9	0.0026	−
10	0.0004	−

Spearman's test: $r = 0.853$, $p = 0.002$.
PD-L1, programmed death-ligand 1; qRT-PCR, quantitative RT polymerase chain reaction; IHC, immunohistochemistry.

Supplementary Table S3: Correlation of CD8+ TILs, FOXP3+ TILs and CD8+/FOXP3+ TILs ratio with PD-L1 expression

	No. of patients %		PD-L1 expression		*P* value
			Negative	Positive	
CD8+ TILs					< 0.001
Low(≤ 55.00)	67	50.4%	17	50	
High(> 55.00)	66	49.6%	60	6	
FOXP3+ TILs					0.054
Low(≤ 21.48)	67	50.4%	33	34	
High(> 21.48)	66	49.6%	44	22	
CD8+/FOXP3+ Ratio					0.067
Low(≤ 2.64)	66	49.6%	33	33	
High(> 2.64)	67	50.4%	44	23	

TILs, tumor-infiltrating lymphocytes; PD-L1, programmed death-ligand 1.

Supplementary Table S4: Correlation of CD8+ TILs, FOXP3+ TILs and CD8+/FOXP3+ ratio with clinicopathological parameters

	**No. patients**	**%**	**CD8+ TILs**			**FOXP3+ TILs**			**Intratumoral CD8+/FOXP3+ Ratio**		
			**Low (≤ 55.00)**	**High (> 55.00)**	***P* value**	**Low (≤ 21.48)**	**High (> 21.48)**	***P*-value**	**Low (≤ 2.64)**	**High (> 2.64)**	***P*-value**
Age (years)											
≤ 59	67	50.4%	30	27	0.193	31	36	0.340	34	33	0.794
> 59	66	49.6%	37	29		36	30		32	34	
Gender											
Male	75	56.4%	37	38	0.784	41	34	0.260	34	41	0.260
Female	58	43.6%	30	28		26	32		32	26	
Pre-op KPS											
KPS ≤ 80	89	66.9%	47	42	0.425	44	45	0.758	48	41	0.158
KPS > 80	44	33.1%	20	24		23	21		18	26	
Tumor location											
Upper	23	17.3%	12	11	0.964	10	13	0.492	13	10	0.185
Middle	57	42.9%	29	28		27	30		32	25	
Lower	53	39.8%	26	27		30	23		21	32	
Tumor length											
≤ 4 cm	70	52.6%	32	38	0.257	37	33	0.546	31	39	0.194
> 4 cm	63	47.4%	35	28		30	33		35	28	
Differential Grade											
Well	46	34.6%	21	25	0.302	21	25	0.660	26	20	0.505
Moderate	53	39.8%	25	28		27	26		24	29	
Poor	34	25.6%	21	13		19	15		16	18	
Histological Type											
Ulcerative type	68	51.1%	39	29	0.242	34	34	0.300	34	34	0.620
Medullary type	47	35.3%	21	26		21	26		25	22	
Fungating type	18	13.5%	7	11		12	6		7	11	
Recurrence											
Yes	110	82.7%	56	54	0.788	53	57	0.360	57	53	0.360
No	23	17.3%	11	12		14	9		9	14	

TILs, Tumor-infiltrating lymphocytes; PD-L1, Programmed death-ligand 1.
